# The TK0271 Protein Activates Transcription of Aromatic Amino Acid Biosynthesis Genes in the Hyperthermophilic Archaeon Thermococcus kodakarensis

**DOI:** 10.1128/mBio.01213-19

**Published:** 2019-09-10

**Authors:** Yasuyuki Yamamoto, Tamotsu Kanai, Tsuyoshi Kaneseki, Haruyuki Atomi

**Affiliations:** a Department of Synthetic Chemistry and Biological Chemistry, Graduate School of Engineering, Kyoto University, Katsura, Nishikyo-ku, Kyoto, Japan; University of Vienna

**Keywords:** *Archaea*, aromatic amino acids, hyperthermophiles, metabolism, physiology, transcription, transcriptional regulation

## Abstract

TrpY from Methanothermobacter thermautotrophicus is a regulator that inhibits transcription of the Trp biosynthesis (*trp*) operon. Here, we show that the TrpY homolog in Thermococcus kodakarensis is not involved in such regulation. There are 87 genes on the T. kodakarensis genome predicted to encode transcriptional regulators (TRs). By screening for TRs that specifically bind to the promoter of the *trp* operon of T. kodakarensis, we identified TK0271. The gene resides in the *aro* operon, responsible for the biosynthesis of chorismate, a precursor for Trp, Tyr, and Phe. TK0271 was expressed in Escherichia coli, and the protein, here designated Tar (*Thermococcales* aromatic amino acid regulator), was purified. Tar specifically bound to the *trp* promoter with a dissociation constant (*K_d_*) value of approximately 5 nM. Tar also bound to the promoters of the Tyr/Phe biosynthesis (*tyr-phe*) and *aro* operons. The protein recognized a palindromic sequence (TGGACA-N_8_-TGTCCA) conserved in these promoters. *In vitro* transcription assays indicated that Tar activates transcription from all three promoters. We cultivated T. kodakarensis in amino acid-based medium and found that transcript levels of the *trp*, *tyr-phe*, and *aro* operons increased in the absence of Trp, Tyr, or Phe. We further constructed a TK0271 gene disruption strain (ΔTK0271). Growth of ΔTK0271 was similar to that of the host strain in medium including Trp, Tyr, and Phe but was significantly impaired in the absence of any one of these amino acids. The results suggest that Tar is responsible for the transcriptional activation of aromatic amino acid biosynthesis genes in T. kodakarensis.

## Abstract (precis)

The mechanisms of transcriptional regulation in archaea are still poorly understood. In this study, we identified a transcriptional regulator in the hyperthermophilic archaeon Thermococcus kodakarensis that activates the transcription of three operons involved in the biosynthesis of aromatic amino acids. The study represents one of only a few that identifies a regulator in *Archaea* that activates transcription. The results also imply that transcriptional regulation of genes with the same function is carried out by diverse mechanisms in the archaea, depending on the lineage.

## INTRODUCTION

Transcription in archaea is initiated by three basal transcription factors, RNA polymerase (RNAP), transcription factor B (TFB), and TATA-binding protein (TBP). The archaeal system resembles the system found in eukaryotes, which utilize RNA polymerase II, transcription factor II B, and TATA-binding protein, respectively, for mRNA synthesis (1). The structure of archaeal RNAP has been elucidated and provides further support that the machineries of the two systems are homologous (2, 3).

Mechanisms of how transcription is regulated in archaea are now attracting much attention (4–6). Studies to identify factors that support and/or regulate transcription are being carried out in a wide range of archaea (4–6). In the methanogens, characterization of the genome-wide occupancy of transcription machinery and its transcriptome in Methanocaldococcus jannaschii revealed that Spt4/5 is a general elongation factor (7). Ptr2 of *M. jannaschii* is a potent transcriptional activator that mediates recruitment of TBP to the *fdxA* promoter (8). HrsM in Methanococcus maripaludis is involved in the regulation of selenium-dependent gene expression (9). EarA regulates transcription of the archaellum operon in *M. maripaludis* (10). In the halophiles, a leucine zipper-type transcriptional activator, GvpE, was identified in Haloferax volcanii that regulates gas vesicle formation genes (11). Also in H. volcanii, XacR activates the transcription of genes related to d-xylose and l-arabinose catabolism (12), and GlpR represses the transcription of fructose metabolic genes (13). TrmB regulates the gluconeogenic production of sugars incorporated into the cell surface S-layer glycoprotein in Halobacterium salinarum (14). Brz is a zinc finger protein in *H. salinarum* that activates transcription of the bacteriorhodopsin gene (15). In the *Thermococcales*, SurR is a global redox regulator that has been studied in a number of members of this order (16–18). Phr (*Pyrococcus* heat shock regulator) is also a regulator in *Thermococcales* that responds to heat shock (19, 20). TFB-RF1 from Pyrococcus furiosus activates transcription of a putative ABC transporter by recruiting TFB to the promoter (21). In the *Crenarchaeota*, BarR, an Lrp-type transcription factor in Sulfolobus acidocaldarius, regulates an aminotransferase gene in response to the presence of β-alanine (22). The Lrs14 member AbfR1 in S. acidocaldarius is involved in the regulation of biofilm formation and motility (23). A TetR-family transcriptional regulator, FadR, is involved in regulation of fatty acid metabolism in S. acidocaldarius (24).

Tryptophan (Trp) biosynthesis requires large amounts of carbon and energy and thus is regulated by a variety of mechanisms in a wide range of microorganisms (25). In *Archaea*, little is known about the mechanisms involved, with the exception of TrpY from Methanothermobacter thermautotrophicus, which regulates transcription of the *trpEGCFBAD* operon in response to the availability of Trp (26). It has clearly been shown that TrpY binds to TRP box sequences located in the overlapping promoter regions between *trpY* and *trpE*. TrpY binding inhibits *trpY* transcription in the absence of Trp and both *trpY* and *trpEGCFBAD* transcription in its presence, acting as a transcriptional repressor.

Thermococcus kodakarensis is a hyperthermophilic archaeon that displays an optimal growth temperature of 85°C (27, 28). The genome sequence is available (29), and a gene disruption system has been developed (30–34), providing the means to study the physiological roles of transcriptional regulators *in vivo*. A number of metabolic pathways that display features distinct from those of bacteria and eukaryotes have been identified in this archaeon, including glycolysis/gluconeogenesis, pentose metabolism, chitin degradation, and coenzyme A biosynthesis (35–43). In contrast, our knowledge on how these pathways are regulated in terms of gene expression is still limited. Concerning transcriptional regulators from T. kodakarensis, Tgr (*Thermococcales* glycolytic regulator) (TK1769) regulates the transcription of genes involved in sugar metabolism (44), while Phr (TK2291) regulates genes involved in the heat shock response (45). SurR is responsible for the regulation of genes involved in hydrogen metabolism in response to the presence of sulfur (18, 46).

Our group has previously characterized the enzymes involved in the biosynthesis of Trp in T. kodakarensis. Biochemical examination of the enzymes revealed the occurrence of regulation via feedback regulation targeting anthranilate synthase (47). The Trp biosynthesis genes are clustered together on the genome (*trpCDEGFBA*) and transcribed as a single RNA of approximately 6,600 bases (48). When cells were cultivated in the presence or absence of Trp, we found that transcript levels were significantly upregulated in the absence of Trp, indicating the presence of regulation at the transcriptional level.

Here, we have examined the machinery and mechanisms involved in the transcriptional regulation of the *trpCDEGFBA* operon in T. kodakarensis. The transcriptional regulator governing the response to the presence/absence of Trp has been identified, along with its binding site. Through both *in vitro* and *in vivo* experiments, we demonstrate that the protein plays a broad role in regulating the transcription of gene clusters involved in the biosynthesis of aromatic amino acids.

## RESULTS

### Examination of a TrpY homolog in T. kodakarensis.

T. kodakarensis harbors a TrpY homolog, which is encoded by TK1227. The TK1227 protein (TkTrpY) is 42% identical to *M. thermautotrophicus* TrpY (MtTrpY). Thus, we examined whether TkTrpY was involved in the transcriptional regulation of the *trp* operon in T. kodakarensis.

**FIG 1:**
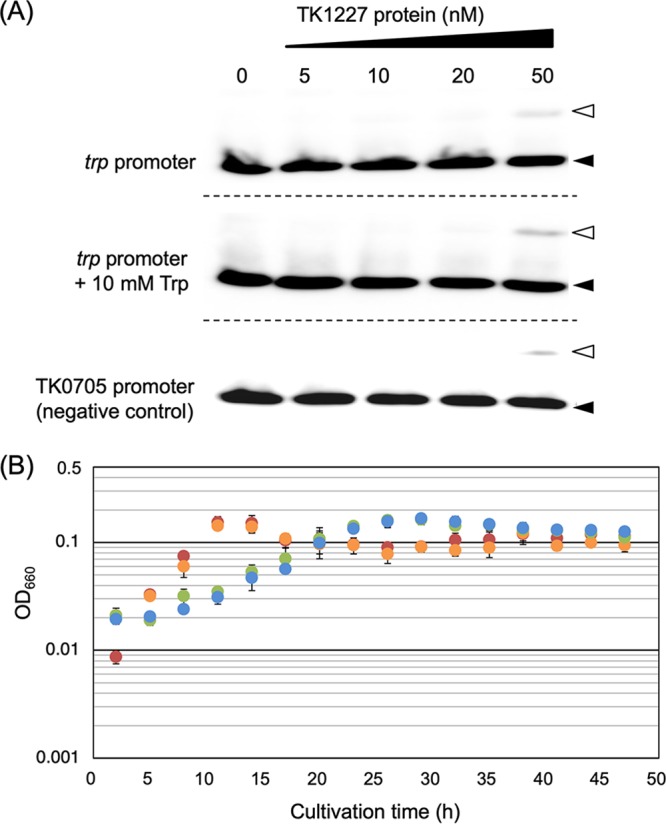
TrpY homolog of T. kodakarensis (TK1227) is not involved in the transcriptional regulation of the *trp* operon. (A) EMSA analysis using various concentrations of the TK1227 protein and the promoter region of the *trp* operon (∼200 bp) and the TK0705 promoter (∼200 bp). In the middle panel, results of EMSA in the presence of Trp (10 mM) are shown. Bands corresponding to free DNA probe and putative protein-DNA complexes are indicated with black and white arrowheads, respectively. (B) Growth curves of the host strain (KU216) in ASW-AA medium with or without Trp (red and orange) and those of the ΔTK1227 strain (KTY1) in ASW-AA medium with or without Trp (green and blue).

The TK1227 gene was expressed in Escherichia coli, and the recombinant protein was purified to apparent homogeneity via incubation at 85°C for 10 min, followed by chromatography using heparin and gel filtration columns. Using the purified TkTrpY (see Fig. S1A in the supplemental material), we carried out electrophoretic mobility shift assays (EMSA) against the promoter region of the *trpCDEGFBA* operon of T. kodakarensis (Fig. 1A). We could not observe specific binding between TkTrpY and the *trp* promoter. When the protein concentration was elevated to 50 nM in the presence of 0.4 nM of the DNA probe, a faint band was observed, but a band was also observed using the promoter of the nonphosphorylating glyceraldehyde-3-phosphate dehydrogenase (GAPN) gene (TK0705). Binding was not affected by the presence or absence of Trp (Fig. 1A).

10.1128/mBio.01213-19.1 FIG S1 (A) SDS-PAGE analysis of recombinant TK1227 protein (TkTrpY) after purification. The gel was stained with Coomassie brilliant blue after electrophoresis. (B) Gene disruption of TK1227. Products of PCR amplification using the genomic DNA of the host strain (KU216) and the ΔTK1227 strain (KTY1) were applied to agarose gel electrophoresis. Primer positions and the expected lengths of the amplified products are shown in the diagram on the right. Download FIG S1, TIF file, 1.8 MB. Copyright © 2019 Yamamoto et al. 2019 Yamamoto et al. This content is distributed under the terms of the Creative Commons Attribution 4.0 International license.

We further constructed a disruption strain of the TK1227 gene (Fig. S1B). Using T. kodakarensis KU216 (31) as a host strain, gene disruption was carried out via single-crossover insertion of the plasmid followed by popout recombination. Cells that had undergone single-crossover insertion were first enriched by growth in a uracil-free medium, and cells that had further undergone popout recombination were selected on solid medium containing uracil and 5-fluoroorotic acid (5-FOA). Transformants displayed lower growth rates than the parent strain, T. kodakarensis KU216. However, growth was not affected upon the presence or absence of Trp (Fig. 1B). The biochemical and genetic analyses both support that the TK1227 protein is not involved in the transcriptional regulation of the *trp* operon in T. kodakarensis.

### A screening system to identify transcriptional regulators.

The results suggested that other factors in T. kodakarensis are involved in the transcriptional regulation of the *trp* operon. In order to screen for proteins that bind to the *trp* promoter, we designed a system based on *in vitro* transcription/translation. By searching the genome sequence data for genes predicted to encode transcriptional regulators, proteins with DNA-binding motifs or proteins with similarity to transcriptional regulators from other organisms/genome sequences, we could estimate that T. kodakarensis harbors at least 87 genes that encode transcriptional regulators (Table S1). We expressed all 87 genes and performed EMSA with a DNA probe including the T. kodakarensis *trp* promoter. Proteins were synthesized *in vitro* with T7 RNA polymerase for transcription and a commercially available translation system based on cell extracts from E. coli. The 87 genes were amplified from genomic DNA, and the T7 promoter and terminator sequences were fused 5′ upstream and 3′ downstream of each gene, respectively. Template DNAs were subjected to *in vitro* transcription/translation, and the reaction mixtures were then incubated at 70°C for 10 min to denature the proteins from E. coli. The constructed library proteins were then used for screening against the *trp* promoter.

10.1128/mBio.01213-19.8 TABLE S1 Putative transcriptional regulators from T. kodakarensis expressed using *in vitro* transcription and translation system. Download Table S1, XLSX file, 0.01 MB. Copyright © 2019 Yamamoto et al. 2019 Yamamoto et al. This content is distributed under the terms of the Creative Commons Attribution 4.0 International license.

In 14 lanes among the 87, we observed either a significant decrease in the intensity of the free probe including the *trp* promoter or a clear shift in mobility (Fig. S2). The genes that were expressed in these lanes were TK0063, TK0142, TK0169, TK0271, TK0888, TK1210, TK1227, TK1272, TK1339, TK1489, TK1881, TK1955, TK2190, and TK2229. We carried out the same experiments with other DNA probes and found that the TK0271 protein was the only protein that displayed specificity to the *trp* promoter. TK0271 was located in the *aro* operon (TK0271-TK0262), which is predicted to be responsible for the biosynthesis of chorismate, a precursor for Trp biosynthesis. Thus, we set out to examine the function of TK0271 in detail.

10.1128/mBio.01213-19.2 FIG S2 EMSA using the *trp* promoter and 87 putative T. kodakarensis transcriptional regulators synthesized by the *in vitro* transcription/translation system. Arrows (blue and red) indicate positions of putative transcriptional regulators showing either a significant decrease in the intensity of the free DNA probe or a clear shift in mobility. Red arrow indicates the position of TK0271. Asterisks indicate nonspecific signals derived from the *in vitro* transcription/translation system. Download FIG S2, TIF file, 2.8 MB. Copyright © 2019 Yamamoto et al. 2019 Yamamoto et al. This content is distributed under the terms of the Creative Commons Attribution 4.0 International license.

### Gene expression of TK0271 and purification of the recombinant protein.

**FIG 2:**
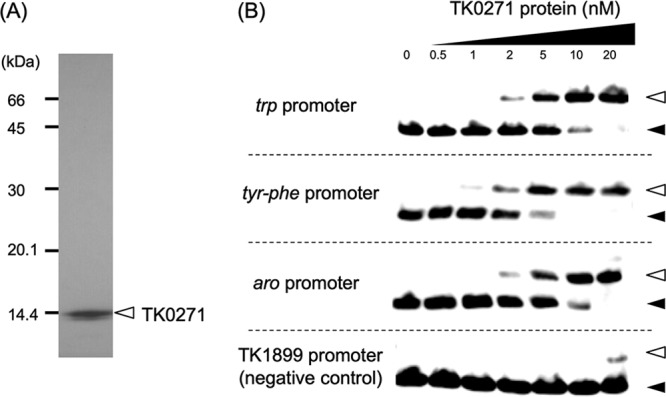
Binding of the TK0271 protein to the promoters of the *trp*, *tyr-phe*, and *aro* operons of T. kodakarensis. (A) SDS-PAGE analysis of recombinant TK0271 protein after purification. The gel was stained with Coomassie brilliant blue after electrophoresis. (B) EMSA analysis using various concentrations of the TK0271 protein and the promoter regions (∼200 bp) of the *trp*, *tyr-phe*, and *aro* operons and the TK1899 (*radA*) promoter. Bands corresponding to free DNA probe and putative protein-DNA complexes are indicated with black and white arrowheads, respectively.

**FIG 3:**
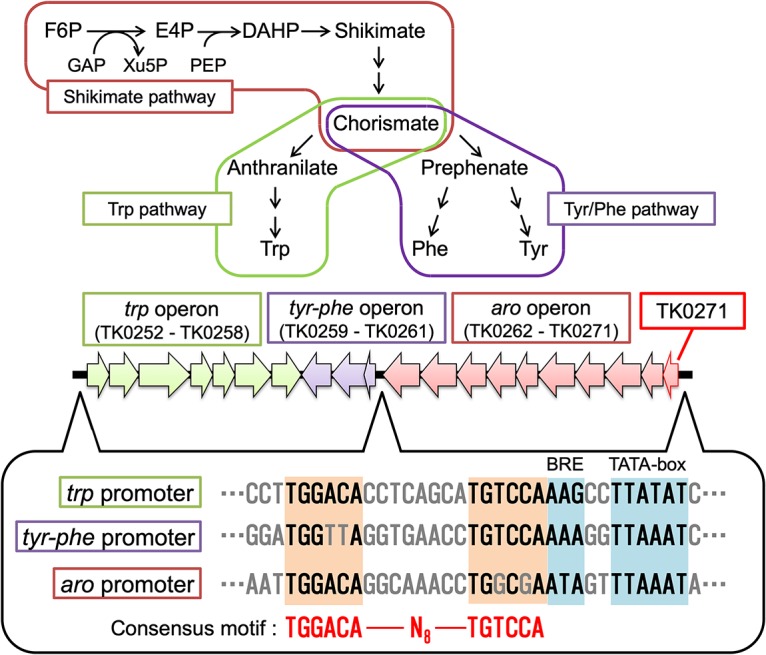
Trp, Tyr, and Phe biosynthesis and gene organization in T. kodakarensis. A simple diagram illustrating the expected biosynthesis pathways for Trp, Tyr, and Phe, and the operons that encode the enzymes, are shown. Abbreviations are the following: F6P, fructose 6-phosphate; E4P, erythrose 4-phosphate; DAHP, 3-deoxy-d-arabinoheptulosonate 7-phosphate; GAP, glyceraldehyde 3-phosphate; Xu5P, xylulose 5-phosphate; and PEP, phosphoenolpyruvate. Below the operons, palindromic sequences found upstream of the putative BRE-TATA sequences in the promoters of each operon are shown.

The TK0271 gene was inserted into the pET21a(+) plasmid and expressed in E. coli BL21-CodonPlus(DE3)-RIL. The soluble protein was purified by heat treatment followed by heparin affinity chromatography and gel filtration chromatography (Fig. 2A). Using this recombinant protein, we carried out EMSA using a DNA fragment containing the *trp* promoter sequence (Fig. 2B). As a result, we could clearly observe shifts in the *trp* probe upon addition of TK0271 protein. Half of the probe population displayed a shift with the addition of 5 nM TK0271 protein, indicating a dissociation constant (*K_d_*) value of approximately 5 nM. As TK0271 was located within the *aro* operon (Fig. 3), we also examined binding using probes corresponding to the *tyr-phe* promoter and *aro* promoter. We found that the TK0271 protein also bound to these promoters with similar affinities (*K_d_* of 5 to 10 nM). We used the promoter sequence of TK1899 (predicted RadA gene) as a control and observed only low levels of mobility shifts at concentrations of 20 nM.

### The binding site of the TK0271 protein.

As the TK0271 protein bound to the *trp*, *tyr-phe*, and *aro* promoters, we searched these promoters for conserved sequences. We observed a candidate palindromic sequence with the consensus TGGACA-N_8_-TGTCCA (Fig. 3). The palindromic sequences were found upstream of the predicted BRE/TATA sequences of each gene promoter.

**FIG 4:**
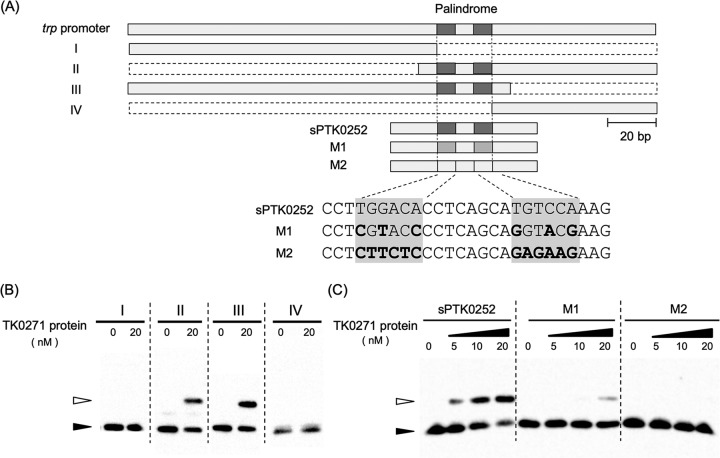
EMSA analysis using the TK0271 protein and DNA fragments of the *trp* promoter. (A) Diagram illustrating the truncated DNA fragments used as probes or those with mutations within the palindromic sequence. (B) Results of EMSA using fragments I to IV. (C) Results of EMSA using fragment sTK0252, M1, or M2. Bands corresponding to free DNA probe and putative protein-DNA complexes are indicated with black and white arrowheads, respectively.

Using the probe for the *trp* promoter, a number of truncated probes were constructed (Fig. 4A, fragments I to IV). The results of EMSA indicated that probes including the palindromic sequence were recognized by the TK0271 protein, whereas those without the sequence were not (Fig. 4B). We further constructed probes with sequence changes within the palindromic sequence. This resulted in a dramatic decrease in affinity of the TK0271 protein with the probes, strongly suggesting that the TK0271 protein recognizes the palindromic sequence (Fig. 4C).

### Construction and characterization of a TK0271 gene disruption strain.

As our *in vitro* experiments suggested the involvement of the TK0271 protein in the transcriptional regulation of the *trp* operon, we constructed a TK0271 gene disruption strain. Using T. kodakarensis KU216 as a host strain, gene disruption was carried out as described above using uracil and 5-FOA. The genotypes of selected transformants were examined by PCR (Fig. S3) and DNA sequencing, indicating that gene disruption had occurred as expected.

10.1128/mBio.01213-19.3 FIG S3 Gene disruption of TK0271. Products of PCR amplification using the genomic DNA of the host strain (KU216) and the ΔTK0271 strain (KAR1) were applied to agarose gel electrophoresis. Primer positions and the expected lengths of the amplified products are shown in the diagram on the right. An asterisk indicates a nonspecific PCR amplification product. Download FIG S3, TIF file, 1.2 MB. Copyright © 2019 Yamamoto et al. 2019 Yamamoto et al. This content is distributed under the terms of the Creative Commons Attribution 4.0 International license.

**FIG 5:**
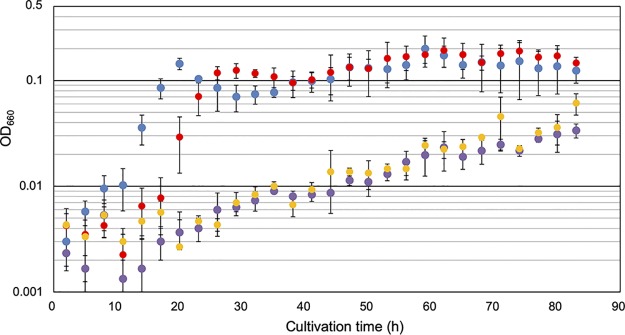
Growth curves of the host strain (KU216) (blue), the ΔTK0271 strain (KAR1) (purple), KAR1 with a plasmid harboring the wild-type TK0271 gene (pTK0271) (red), and KAR1 with an empty plasmid (pLC70Δ) (yellow) grown in medium without Trp. Specific growth rates were 0.31 h^−1^, 0.028 h^−1^, 0.26 h^−1^, and 0.037 h^−1^, respectively.

The growth of the TK0271 gene disruption strain (KAR1), along with the parent host strain T. kodakarensis KU216, was examined in a synthetic medium with or without Trp. As shown in Fig. 5, we observed a significant retardation in growth of KAR1 cells compared to that of KU216 cells in medium without Trp. The growth impairment was not observed when Trp was added to the medium (Fig. S4), suggesting that TK0271 is involved in Trp biosynthesis. When an intact TK0271 gene was introduced to the KAR1 strain in *trans* on a replicating plasmid, the growth retardation was no longer apparent, indicating that the growth defect in medium without Trp was not due to polar effects and solely due to the absence of the TK0271 gene. We next grew the two strains in medium without Phe or Tyr. Surprisingly, the effects of TK0271 gene disruption were even greater. Although we observed low levels of growth in medium without Trp, we could not observe any growth of KAR1 cells in medium without Phe or Tyr. The results indicate that the function of TK0271 contributes to the biosynthesis of Trp, Phe, and Tyr in T. kodakarensis.

10.1128/mBio.01213-19.4 FIG S4 Growth curves of the host strain (KU216) (blue) and the ΔTK0271 strain (KAR1) (purple) in a synthetic medium (ASW-AA; including Trp) containing elemental sulfur (2 g liter^−1^), sodium pyruvate (5 g liter^−1^), maltodextrin (5 g liter^−1^), and 0.1 mM Na_2_WO_4_. Download FIG S4, TIF file, 1.0 MB. Copyright © 2019 Yamamoto et al. 2019 Yamamoto et al. This content is distributed under the terms of the Creative Commons Attribution 4.0 International license.

### Transcriptome analyses.

**FIG 6:**
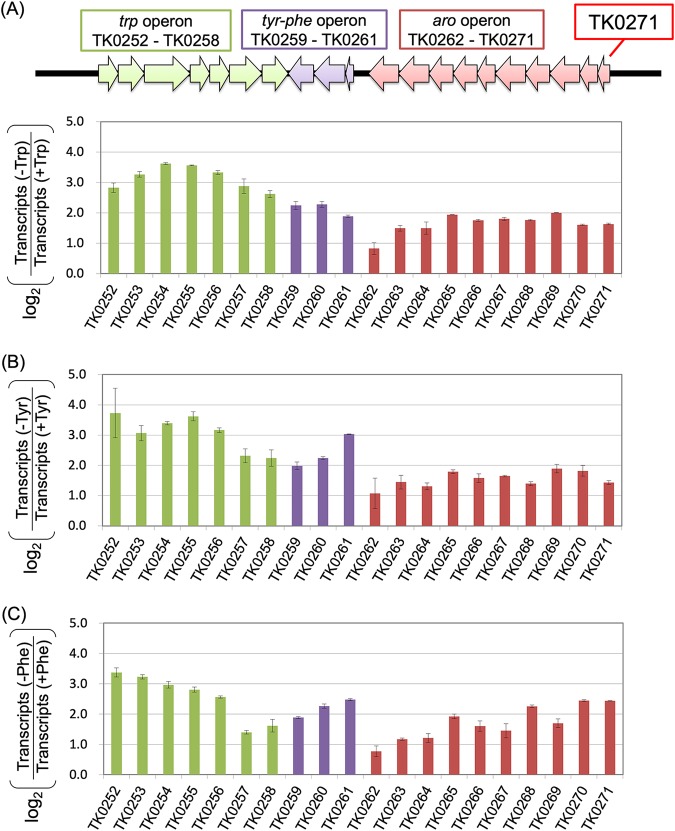
Relative transcript levels of the *trp*, *tyr-phe*, and *aro* operon genes in response to the presence or absence of Trp (A), Tyr (B), or Phe (C). The operons are shown in panel A. The results are those obtained with the host strain T. kodakarensis KU216.

We further performed transcriptome analyses on KU216 cells grown in the presence or absence of Trp, Phe, or Tyr. Intriguingly, we found that transcript levels of the *trp*, *tyr-phe*, and *aro* gene clusters were all higher when cells were grown in the absence of any one of these amino acids, e.g., the absence of Phe leads to upregulation of the *trp* operon genes (Fig. 6A to C). Together with the observation that TK0271 protein binding sites were present in the promoter regions of all three gene clusters, the results of our transcriptome studies strongly suggest that the transcription of the *trp*, *tyr-phe*, and *aro* gene clusters are regulated by a single common mechanism involving TK0271.

### TK0271 protein is a transcriptional activator.

**FIG 7:**
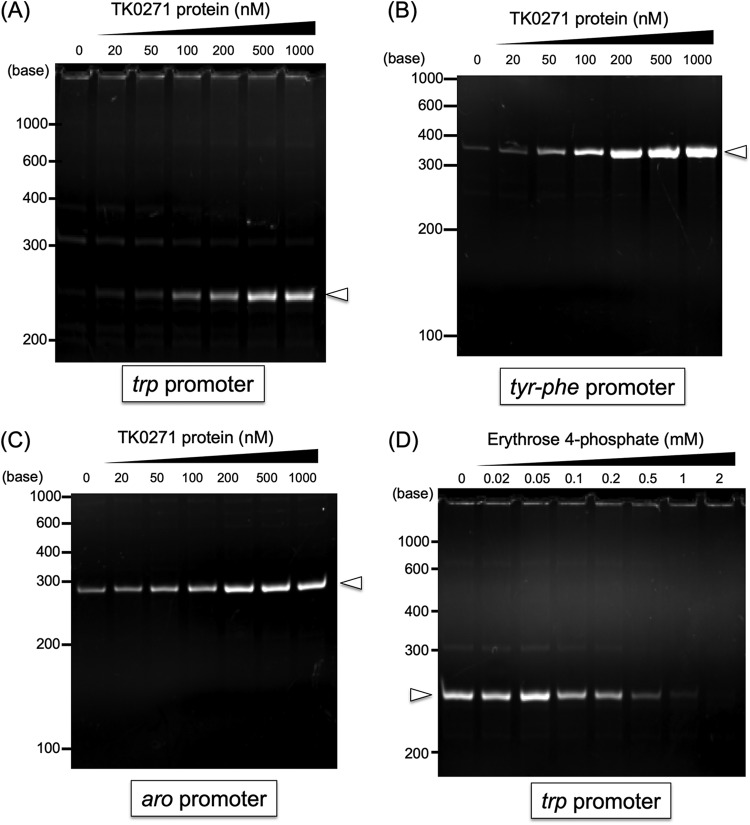
Effects of the TK0271 protein on *in vitro* transcription analysis using TBP (300 nM), TFB (300 nM), and RNAP (200 nM) from T. kodakarensis. The promoters used were from the *trp* operon (A), the *tyr-phe* operon (B), and the *aro* operon (C). The concentration of the DNA fragments containing the promoters was 50 nM, and the TK0271 protein was added at concentrations of 0, 20, 50, 100, 200, 500 and 1,000 nM. After the reactions and electrophoresis, gels were stained with ethidium bromide (see Materials and Methods for details). The expected RNA lengths are 250 bases, 360 bases, and 286 bases for the *trp* operon, the *tyr-phe* operon, and the *aro* operon, respectively. (D) Effects of erythrose 4-phosphate on *in vitro* transcription activated by the TK0271 protein. The TK0271 protein was added at a concentration of 1,000 nM, and the concentrations of erythrose 4-phosphate were 0, 0.02, 0.05, 0.1, 0.2, 0.5, 1, and 2 mM.

As growth in medium depleted of Trp, Tyr, or Phe was impaired upon gene disruption of TK0271, the TK0271 protein can be presumed to have a positive effect on the expression of the biosynthesis genes. Binding sites of the TK0271 protein are found upstream of the BRE/TATA box, which is also the case for other transcriptional regulators identified in archaea that act as activators (8, 21, 44). In order to examine whether the TK0271 protein is a transcriptional activator, we carried out *in vitro* transcription on a DNA template corresponding to the *trp* promoter region and a portion of the *trpC* gene, the first gene in the operon. Transcription was performed in the presence of the basal transcription factors from T. kodakarensis, RNA polymerase, TFB, and TBP, with or without the TK0271 protein. In the absence of TK0271 protein, we could detect only faint levels of transcripts (Fig. 7A). When we added the TK0271 protein and gradually increased its concentration, we observed a significant increase in transcript levels. Similar increases in transcript levels were also obtained with *tyr-phe* (Fig. 7B) and *aro* (Fig. 7C) promoters by the addition of the TK0271 protein. These results confirm that the TK0271 protein is a transcriptional activator of the *trp*, *tyr-phe*, and *aro* operons (Fig. 3) in T. kodakarensis. Thus, the TK0271 protein was designated Tar, for *Thermococcales* aromatic amino acid regulator.

### Search for effector molecules that affect Tar binding.

**FIG 8:**
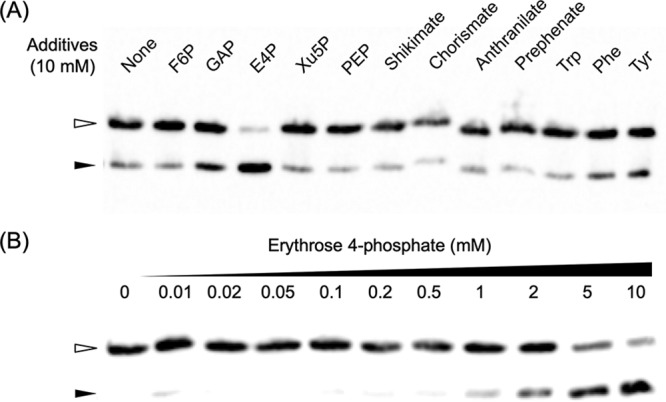
Effects of various metabolites on the binding of the TK0271 protein to the *trp* promoter. (A) EMSA was carried out using 0.4 nM DNA probe and 20 nM the TK0271 protein. Fructose 6-phosphate (F6P), glyceraldehyde 3-phosphate (GAP), erythrose 4-phosphate (E4P), xylulose 5-phosphate (Xu5P), phosphoenolpyruvate (PEP), shikimate, chorismite, anthranilate, prephenate, Trp, Phe, or Tyr was added at a concentration of 10 mM. (B) EMSA was carried out as described for panel A but with various concentrations of erythrose 4-phosphate.

Our results have clearly demonstrated that Tar binds to a palindromic sequence in the promoter regions of the *aro*, *trp*, and *tyr-phe* gene clusters and activates transcription. We next examined whether particular metabolites would trigger the release of Tar from its binding site. Primary candidates were the amino acids themselves (Trp, Tyr, and Phe), but we also examined other compounds in the biosynthesis pathways of each amino acid. These were shikimate, chorismate, anthranilate, prephenate, fructose 6-phosphate (F6P), glyceraldehyde 3-phosphate (GAP), erythrose 4-phosphate (E4P), xylulose 5-phosphate (Xu5P), and phosphoenolpyruvate (PEP). As shown in Fig. 8A, we did not observe the release of the DNA probe when Trp, Tyr, and Phe were added at concentrations of 10 mM. However, we observed a significant release of the probe in the presence of E4P. When we examined this effect at different concentrations of E4P, we found that the majority of the Tar-DNA complex dissociated at 5 mM E4P (Fig. 8B). The effect of E4P was further supported by the observation that the addition of E4P to the *in vitro* transcription system where transcription was activated by the Tar protein resulted in a decrease in transcription using the *trp* promoter (Fig. 7D).

## DISCUSSION

### Distribution of Tar homologs.

We examined the distribution of Tar (TK0271) homologs and found that closely related homologs (>60% identical) are found in a number of, but not all, members of the *Themococcales*. Those harboring Tar homologs include Thermococcus onnurineus, Thermococcus gammatolerans, Thermococcus litoralis, Pyrococcus furiosus, and *Pyrococcus abyssi*, and a phylogenetic analysis of the proteins is shown in Fig. S5 in the supplemental material. Genome sequences indicate that the presence of a Tar homolog is linked with the presence of an *aro* gene cluster and not necessarily with *trp* operons. Most strikingly, the Tar homolog genes are all included within the *aro* operons. As the genomes with Tar homologs and *aro* operons also harbor one or more aromatic amino acid biosynthesis gene clusters (*trp* and *tyr-phe*), it can be presumed that these Tar homologs are also involved in the regulation of aromatic amino acid biosynthesis. Indeed, we observed the presence of sequences closely related to the TGGACA-N_8_-TGTCCA consensus sequence identified in T. kodakarensis. Although with lower similarity, *P. abyssi* harbors an additional TK0271 homolog on its genome, but this gene forms a cluster with genes annotated as components of a tungsten-containing oxidoreductase and may be involved in the regulation of these genes. Other than the *Thermococcales*, homologs 31 to 45% identical to Tar are found in a limited number of genomes from archaea (*Korarchaeum*) and bacteria (Methylocella silvestris) (Fig. S5). As these genes do not reside within *aro* operons, we cannot predict their functions. The homolog from *M. silvestris* is considered a member of the MarR family and is predicted to have a helix-turn-helix (HTH) domain. This raises the possibility that Tar and its homologs also contain an HTH domain.

10.1128/mBio.01213-19.5 FIG S5 Phylogenetic tree of TK0271 homolog sequences from *Archaea* and *Bacteria*. Proteins are from Thermococcus kodakarensis (TK0271), Thermococcus litoralis (OCC_05581), *Thermococcus* sp. strain 2319x1 (ADU37_CDS10110), *Thermococcus onnurineus* (TON_1130), Thermococcus chitonophagus (CHITON_1974), Thermococcus celer (A3L02_04690), Thermococcus paralvinellae (TES1_0480), Thermococcus sibiricus (TSIB_1603), Thermococcus barophilus (TERMP_00417), Thermococcus gammatolerans (TGAM_1601), Pyrococcus furiosus DSM 3638 (PF1687), *Pyrococcus abyssi* (PAB2083 and PABs3696), Pyrococcus horikoshii (PH1714.1n), *Pyrococcus* sp. strain ST04 (Py04_1631), *Pyrococcus* sp. strain NA2 (TQ32_08200), *Pyrococcus* sp. strain NA2 (PNA2_0324), “*Candidatus* Korarchaeum cryptofilum” (Kcr_0130), and Methylocella silvestris (Msil_3155). Only bootstrap values above 50% are indicated. The proteins from T. kodakarensis (TK0271) and *P. abyssi* (PAB2083) that are discussed in the text are indicated with asterisks. Download FIG S5, TIF file, 1.2 MB. Copyright © 2019 Yamamoto et al. 2019 Yamamoto et al. This content is distributed under the terms of the Creative Commons Attribution 4.0 International license.

### Transcriptional regulation of the *aro*, *trp*, and *tyr-phe* operons in T. kodakarensis.

Based on our results, it can be presumed that Tar is a transcriptional activator that enhances the transcription of the *aro*, *trp*, and *tyr-phe* operons in T. kodakarensis. Intriguingly, Tar binding is not affected by the addition of chorismate, Trp, Tyr, or Phe, the final products of the biosynthesis pathways encoded by these operons. Release of Tar was observed in the presence of E4P. One possibility that might explain the effect of E4P is the involvement of 3-deoxy-d-arabinoheptulosonate-7-phosphate (DAHP) synthase (DAHPS). DAHPS catalyzes the second reaction in the biosynthesis of chorismate and utilizes E4P as the substrate. In many microorganisms, such as E. coli and Saccharomyces cerevisiae, it has been reported that DAHPS is inhibited by aromatic amino acids (49). If this were the case in T. kodakarensis, this would provide a feasible explanation for the effect of E4P on Tar binding. High concentrations of Trp would inhibit the reaction of DAHPS by feedback inhibition, leading to an increase in E4P concentration. E4P would then bind to Tar, which releases the protein from the promoter, resulting in the loss of activation of the regulated operons. However, it has been shown that the DAHPS from P. furiosus, which is 83.2% identical to that from T. kodakarensis, is not inhibited by Trp, Tyr, or Phe (50). The DAHPS from the hyperthermophilic archaeon Aeropyrum pernix also is not regulated by these amino acids (51). The DAHPS from P. furiosus and *A. pernix* do not harbor N- or C-terminal extension regions that are involved in feedback inhibition of DAHPS (49). On the other hand, the DAHPS from T. kodakarensis harbors an extra C-terminal extension composed of around 40 amino acids (Fig. S6, underlined residues). Moreover, DAHPSs from E. coli and S. cerevisiae also contain a C-terminal extension with a length of ∼40 residues. Although there are no sequence similarities between these regions and that of T. kodakarensis, this extension may be involved in the feedback regulation of DAHPS in T. kodakarensis. However, we cannot rule out the possibilities of other compounds triggering the release of Tar from its binding region.

10.1128/mBio.01213-19.6 FIG S6 Multiple alignments of DAHPS from T. kodakarensis (TK0268), P. furiosus (PF1690), *A. pernix* (APE_0581.1), E. coli (b0754, b1704, and b2601), and Saccharomyces cerevisiae (YBR249C and YDR035W). Asterisks indicate conserved amino acid residues. The C-terminal extension region of TK0268 is underlined. Download FIG S6, PDF file, 0.02 MB. Copyright © 2019 Yamamoto et al. 2019 Yamamoto et al. This content is distributed under the terms of the Creative Commons Attribution 4.0 International license.

### Lack of specificity in regulation.

Our results indicate that the transcription of the *aro*, *trp*, and *phe*/*tyr* operons is regulated through a common mechanism. The absence of one of these amino acids triggers the upregulation of all three operons. It is difficult to imagine what advantages are provided with a regulation mechanism with such a lack of specificity. This may reflect that there are very few situations in native environments in which only one of the three amino acids is absent. On the other hand, it is also interesting how T. kodakarensis recognizes the absence of any one of the three amino acids.

### Distribution of TrpY homologs.

Our results, together with those of a study on *M. thermautotrophicus*, indicate that TrpY homologs display distinct functions depending on the organism. We now know that MtTrpY from *M. thermautotrophicus* is involved in the transcriptional regulation of the *trp* operon, whereas TkTrpY from T. kodakarensis is not. In order to examine whether function can be distinguished by primary structure, we performed a BLAST search using the amino acid sequence of TK1227 and constructed a phylogenetic tree using 105 homologous sequences from various archaea (Fig. S7). The tree clearly indicates that TrpY homologs can be divided into three or more groups, and that those from the *Thermococcales* cluster in a branch distinct from that including MtTrpY and homologs from other methanogens. This supports the indications that TkTrpY is not involved in the regulation of the *trp* operon in T. kodakarensis and displays a function distinct from that of MtTrpY in *M. thermautotrophicus*. We observed another one or two branches which include homologs from methanogens, haloarchaea, and members of the *Archaeoglobaceae*, but members of these branches have not been examined and we cannot estimate their functions.

10.1128/mBio.01213-19.7 FIG S7 Phylogenetic tree of TK1227 homolog sequences from *Archaea*. Proteins are from Thermococcus kodakarensis (TK1227), Thermococcus peptonophilus (A0127_08790), Thermococcus gorgonarius (A3K92_05310), *Thermococcus radiotolerans* (A3L10_03350), *Thermococcus* sp. strain 5-4 (CDI07_03145), Thermococcus barossii (A3L01_03215), *Thermococcus onnurineus* (TON_0499), Thermococcus piezophilus (A7C91_08515), Thermococcus thioreducens (A3L14_04890), Thermococcus siculi (A3L11_07165), Thermococcus nautili (BD01_0401), Thermococcus profundus (A3L09_07715), Thermococcus eurythermalis (TEU_08505), Thermococcus cleftensis (CL1_0189), Thermococcus guaymasensis (X802_06900), Thermococcus gammatolerans (TGAM_0702), Thermococcus pacificus (A3L08_06235), Pyrococcus furiosus COM1 (PFC_07090), *Thermococcus* sp. strain 4557 (GQS_02705), *Thermococcus* sp. strain AM4 (TAM4_631), Thermococcus celer (A3L02_02560), Thermococcus paralvinellae (TES1_0878), Pyrococcus furiosus DSM 3638 (PF1572), Thermococcus barophilus (TERMP_00866), Pyrococcus yayanosii (PYCH_03810), *Thermococcus* sp. strain P6 (A3L12_05650), Pyrococcus horikoshii (PH1554), *Pyrococcus abyssi* (PAB2435), *Thermococcus* sp. strain 2319x1 (ADU37_CDS20470), *Pyrococcus* sp. strain NA2 (PNA2_0140), *Pyrococcus* sp. strain ST04 (Py04_1448), Thermococcus litoralis (OCC_05294), Thermococcus chitonophagus (CHITON_1795), Pyrococcus kukulkanii (TQ32_09075), Palaeococcus pacificus (PAP_07805), Thermococcus sibiricus (TSIB_1918), *Methanobacterium* sp. strain MZ-A1 (BK008_07080), Methanobacterium subterraneum (BK009_11505), *Methanobrevibacter* sp. strain AbM4 (Abm4_0245), Methanobacterium congolense (MCBB_0597), Methanobacterium lacus (Metbo_2049), Methanobacterium paludis (MSWAN_0589), Methanobacterium formicicum DSM1535 (DSM1535_0197), Methanobacterium formicicum BRM9 (BRM9_2005), *Methanobzacterium* sp. strain BAmetb5 (CIT02_05535), *Methanobacterium* sp. strain MB1 (MBMB1_0275), Methanobrevibacter ruminantium (mru_0477), Methanobrevibacter olleyae (YLM1_0151), *Methanothermobacter* sp. strain MT-2 (METMT2_1486), Methanothermobacter marburgensis (MTBMA_c02330), Methanothermus fervidus (Mfer_0099), Methanococcoides burtonii (Mbur_1076), *Methanobacterium* sp. strain BRmetb2 (CIT01_06920), Methanococcoides methylutens (MCMEM_1599), Methanothermobacter wolfeii (MWSIV6_0193), Methanobrevibacter smithii (Msm_0635), Methanosphaera stadtmanae (Msp_1069), *Methanobrevibacter* sp. strain YE315 (TL18_01445), Ferroglobus placidus (Ferp_0114), *Methanothermobacter* sp. strain EMTCatA1 (Metev_0800), Methanosarcina mazei C16 (MSMAC_2121), Methanosarcina mazei S-6 (MSMAS_2324), Methanosarcina mazei Tuc01 (MmTuc01_2427), Methanosarcina mazei Go1 (MM_2375), Geoglobus ahangari (GAH_00853), Archaeoglobus profundus (Arcpr_0372), Methanothrix thermoacetophila (Mthe_0699), Archaeoglobus fulgidus DSM 8774 (AFULGI_00011160), Archaeoglobus fulgidus DSM 4304 (AF_1020), Methanohalophilus halophilus (BHR79_00510), Methanobrevibacter millerae (sm9_0337), Methanothermobacter thermautotrophicus (MTH_1654), Geoglobus acetivorans (GACE_1783), Halogeometricum borinquense (Hbor_02610), *Methanothermobacter* sp. strain CaT2 (MTCT_1509), Archaeoglobus veneficus (Arcve_1454), Methanosaeta harundinacea (Mhar_1186), *Methanothermobacter* sp. strain EMTCatA1 (tca_01602), Methanosarcina acetivorans (MA_1396), Methanosalsum zhilinae (Mzhil_0636), Methanosarcina horonobensis (MSHOH_2928), Archaeoglobus sulfaticallidus (Asulf_01955), *Methanosarcina* sp. strain MTP4 (MSMTP_2187), *Methanosphaera* sp. strain BMS (AW729_10545), Methanohalophilus mahii (Mmah_0580), Haloferax mediterranei (HFX_2978), Methanothrix soehngenii (MCON_0905), Methanosarcina thermophila TM-1 (MSTHT_0830), Methanosarcina thermophila CHTI-55 (MSTHC_2441), *Methanolobus psychrophilus* (Mpsy_1027), Methanosarcina lacustris (MSLAZ_0760), Ferroplasma acidiphilum (FAD_1150), *Ferroplasma acidarmanus* (FACI_IFERC01G0234), Haloferax gibbonsii (ABY42_14880), Salinigranum rubrum (C2R22_20680), Haloquadratum walsbyi C23 (Hqrw_1030), Haloferax volcanii (HVO_2984), Haloquadratum walsbyi DSM 16790 (HQ_1027A), Methanocella conradii (Mtc_1746), *Natronolimnobius* sp. strain AArc1 (AArc1_0385), *Natronolimnobius* sp. strain AArc-Mg (AArcMg_0377), Haloterrigena turkmenica (Htur_0109), Methanosarcina flavescens (AOB57_012350), Methanocella arvoryzae (LRC278), Methanosarcina barkeri Wiesmoor (MSBRW_2572), and Methanosarcina barkeri Fusaro (Mbar_A2169). Only bootstrap values above 50% are indicated. The proteins from T. kodakarensis (TK1227) and *M. thermautotrophicus* (MTH_1654) that are discussed in the text are indicated with asterisks. Download FIG S7, TIF file, 0.8 MB. Copyright © 2019 Yamamoto et al. 2019 Yamamoto et al. This content is distributed under the terms of the Creative Commons Attribution 4.0 International license.

### Diversity in transcriptional regulation mechanisms among the archaea.

Our results clearly demonstrate that T. kodakarensis (via Tar) and *M. thermautotrophicus* (via TrpY) utilize distinct mechanisms for the transcriptional regulation of the *trp* operon. Interestingly, although many members of the *Crenarchaeota* harbor *aro*, *tyr-phe*, and/or *trp* operons, almost none of their genomes harbor Tar homologs or TrpY homologs. This raises the possibility that *Crenarchaeota* utilize a third regulator or mechanism for regulation of these genes. This suggests that the mechanisms for transcriptional regulation of genes with the same function is diverse among the archaea, depending on their phylogeny.

## MATERIALS AND METHODS

### Strains and culture conditions.

E. coli DH5α was used for routine DNA manipulation. E. coli BL21-CodonPlus(DE3)-RIL (Stratagene, La Jolla, CA) was used for heterologous gene expression. E. coli strains were cultivated at 37°C in lysogeny broth (LB) medium (10 g liter^−1^ tryptone, 5 g liter^−1^ yeast extract, 5 g liter^−1^ NaCl, pH 7.0) containing 100 μg ml^−1^ ampicillin. T. kodakarensis KU216 (31) and its derivative strains were cultivated under anaerobic conditions at 85°C in a nutrient-rich medium (artificial seawater with yeast extract and tryptone [ASW-YT]) or a synthetic medium (ASW with amino acids [ASW-AA]) as described previously (30). Uracil (Ura; 5 mg liter^−1^), 5-fluoroorotic acid (5-FOA; 7.5 g liter^−1^), elemental sulfur (S^0^; 0.2 g liter^−1^), sodium pyruvate (Pyr; 5 g liter^−1^), and/or maltodextrin (Mdx; 5 g liter^−1^) were added when necessary.

### *In vitro* protein synthesis.

In order to prepare template DNA for *in vitro* protein synthesis, two steps of PCR were used. In the first step, each open reading frame (ORF) was amplified with common tag sequences for the next PCR, 5′-TAAGAAGGAGATATACCATG-3′ for the upstream region (initiation codon is underlined) and 5′-TAACTAACTAAGCCACCGCT-3′ for the downstream region. The sequences of the primers are shown in Table S2 in the supplemental material. Amplification was confirmed for each PCR solution by agarose gel electrophoresis. In the second step, the PCR solutions were used as templates without purification. Common primers for the second PCR include sequences needed for *in vitro* protein synthesis (T7 promoter and T7 terminator) (Table S2). The second PCR solutions were used for *in vitro* protein synthesis reaction without DNA purification.

10.1128/mBio.01213-19.9 TABLE S2 Primers to amplify template DNA for *in vitro* protein synthesis. Download Table S2, XLSX file, 0.01 MB. Copyright © 2019 Yamamoto et al. 2019 Yamamoto et al. This content is distributed under the terms of the Creative Commons Attribution 4.0 International license.

For *in vitro* protein synthesis, a protein synthesis system that utilizes E. coli cell extract (RTS 100 E. coli HY kit; biotechrabbit GmbH, Hennigsdorf, Germany) was used with a standard protocol. The protein synthesis reaction was performed at 30°C for 6 h in a 25-μl reaction solution that included 5 μl of template DNA solution. After the reaction, solutions were incubated at 70°C for 10 min and centrifuged at 20,400 × *g* for 30 min to remove thermolabile proteins from the E. coli cell extract.

### Expression of the TK1227 and TK0271 genes and purification of the recombinant proteins.

The TK1227 and TK0271 genes were amplified by PCR from genomic DNA using primers with extensions with an NdeI or BamHI site (TK0271_F/TK0271_R and TK1227_F/TK1227_R). Note that sequences of all primers used here are shown in Table S3. The amplified DNA fragments were digested with NdeI and BamHI and inserted into pET21a(+) (Novagen, Madison, WI). After individually introducing the plasmids into E. coli BL21-CodonPlus(DE3)-RIL, cells were grown until the optical density at 660 nm (OD_660_) exceeded 0.4, and gene expression was induced by the addition of 0.5 mM isopropyl-1-thio-β-d-galactopyranoside (IPTG). After a further 4 h of incubation at 37°C, cells were harvested by centrifugation (5,000 × *g*, 15 min, 4°C). Cells were resuspended in 10 mM sodium phosphate buffer (pH 7.5) and disrupted by sonication at 0°C. After centrifugation (20,400 × *g*, 30 min, 4°C), the supernatants were incubated at 85°C for 10 min and centrifuged (20,400 × *g*, 30 min, 4°C) to remove thermolabile proteins from E. coli. Supernatants after heat treatment were purified by a heparin affinity column (HiTrap Heparin HP; GE Healthcare, Chicago, IL). The initial buffer was 10 mM sodium phosphate (pH 7.5), and proteins were eluted with 10 mM sodium phosphate (pH 7.5), 1 M NaCl. Relevant fractions were collected and concentrated with Amicon Ultra 10K (Merck, Darmstadt, Germany) and subjected to a gel filtration column (Superdex 75 10/30 GL; GE Healthcare) with 50 mM Tris-HCl (pH 7.5), 150 mM NaCl as the mobile phase.

10.1128/mBio.01213-19.10 TABLE S3 Other primers used in this study. Download Table S3, XLSX file, 0.01 MB. Copyright © 2019 Yamamoto et al. 2019 Yamamoto et al. This content is distributed under the terms of the Creative Commons Attribution 4.0 International license.

### EMSA.

Biotinylated probes for EMSA were prepared either by PCR or annealing of two complementary oligonucleotides using a 5′-biotinylated primer. For the genes TK0252 (*trp*), TK0261 (*tyr-phe*), TK0271 (*aro*), TK0705, and TK1899, an ∼200-bp promoter region containing BRE-TATA sequences together with a 10- to 15-bp portion of the ORF was amplified with the primer sets PTK0252_F/PTK0252_R_bt, PTK0261_F/PTK0261_R_bt, PTK0271_F/PTK0271_R_bt, PTK0705_F/PTK0705_R_bt, and PTK1899_F/PTK1899_R_bt, respectively. One primer in each primer set was biotinylated (one with an abbreviation of “bt”). For truncated regions of the TK0252 promoter, the primer sets used for amplification were PTK0252_F_bt/PTK0252_R2 (for fragment I), PTK0252_F1/PTK0252_R_bt (for fragment II), PTK0252_F_bt/PTK0252_R1 (for fragment III), and PTK0252_F2/PTK0252_R_bt (for fragment IV). For DNA fragments with mutations in the consensus sequence (TGGACA-N_8_-TGTCCA) of the TK0252 promoter, two complementary oligonucleotides with the mutated sequences were annealed and overhangs were filled with KOD plus DNA polymerase. The primer sets sPTK0252_bt/sPTK0252, sPTK0252_bt/sPTK0252m1, and sPTK0252_bt/sPTK0252m2 were used for preparing the wild-type, M1, and M2 fragments, respectively. These amplified DNA fragments were purified using a QIAquick gel extraction kit (Qiagen, Hilden, Germany). For EMSA, a 4-μl aliquot of the *in vitro* protein synthesis reaction solution or purified transcriptional regulator was incubated with 0.4 nM biotinylated probe DNA at 70°C for 10 min in 20 μl of binding solution (10 mM Tris-HCl [pH 7.5], 1 mM dithiothreitol [DTT], 10 mM MgCl_2_, 400 mM KCl, and 0.05% NP-40). After incubation, samples were loaded onto a 5% polyacrylamide gel. Gels were run with 0.5× Tris-borate EDTA (TBE) buffer at 100 V for 90 min. After electrophoresis, gels were blotted onto nylon membranes (Hybond-N+; GE Healthcare) at 200 mA for 30 min. In order to cross-link nylon membrane and transferred DNA, the membrane was incubated at 85°C for 1 h. Probes were visualized using a LightShift chemiluminescent EMSA kit (Thermo Fisher Scientific, Waltham, MA). Effects of metabolites (each 10 mM) on the binding of the TK0271 protein to the *trp* promoter were analyzed using 0.4 nM DNA probe and 20 nM the TK0271 protein. EMSA was also carried out with various concentrations of erythrose 4-phosphate from 0 to 10 mM.

### Production of recombinant TBP and TFB proteins in E. coli and their purification.

Genes encoding TBP (TK0132) and TFB (TK1280) were amplified from T. kodakarensis KOD1 genomic DNA using the primer sets TBP-1/TBP-2 for the TBP gene and TFB-1/TFB-2 for the TFB gene. These amplified fragments were digested with NdeI and BamHI and inserted into the respective sites of pET21a(+), resulting in the expression plasmids pET-TBP and pET-TFB. Gene expression in E. coli BL21-CodonPlus(DE3)-RIL was performed as described for TK1227 and TK0271 genes. In the case of recombinant TBP, cell disruption and heat treatment were performed with methods similar to those applied for TK1227 and TK0271 proteins. The supernatant after heat treatment and centrifugation was applied to anion-exchange chromatography using a HiTrap Q HP column (GE Healthcare). Proteins were eluted with Tris-HCl buffer (50 mM, pH 8.0) and an NaCl gradient (0 to 1.0 M). The eluted samples were applied to a Superdex 200 HR 10/30 column (GE Healthcare) equilibrated with 50 mM Tris-HCl (pH 8.0), 150 mM NaCl. In the case of TFB, cells were suspended in 10 mM sodium phosphate (pH 7.0) and lysed by sonication. The supernatant after centrifugation was applied to a 5-ml HiTrap Heparin HP column, and TFB was eluted with 10 mM sodium phosphate buffer (pH 7.0), 1.2 M NaCl. After addition of ammonium sulfate to a final concentration of 2.0 M and centrifugation at 5,000 × *g* for 10 min, the precipitate was suspended in 10 mM sodium phosphate buffer (pH 7.0) containing 100 mM KCl and 400 mM (NH_4_)_2_SO_4_. Salts were removed with a HiPrep desalting 26/10 column (GE Healthcare) equilibrated with 50 mM Tris-HCl buffer (pH 8.0), 100 mM KCl.

### Preparation of T. kodakarensis RNAP with a His_6_ tag on subunit L.

T. kodakarensis RNA polymerase (RNAP) was prepared with T. kodakarensis KUWL (52). In this strain, the chromosomal copy of the *rpoL* gene, encoding subunit L, was replaced by a *rpoL-his*_6_ allele in which the ORF is extended by six histidine codons. This strain was grown at 85°C in ASW-YT-Pyr until the late exponential growth phase (OD of 0.65). After centrifugation, cells were suspended in 20 mM sodium phosphate buffer (pH 7.4) containing 2 M KCl and 0.1 M DTT. Cells were disrupted with a French press (FA-003; Thermo Electron Co.) twice with a pressure of 20,000 lb/in^2^. After centrifugation (38,500 × *g*, 30 min), a 30% (wt/vol) polyethylene glycol 8000 solution was added to the supernatant to a final concentration of 6%. After centrifugation at 38,500 × *g* for 75 min, the supernatant was diluted 20-fold and adjusted to 20 mM sodium phosphate buffer (pH 7.4) containing 0.5 M KCl and 20 mM imidazole. The solution was applied to a nickel column (HisTrap HP; GE Healthcare), and RNAP proteins were eluted using 20 mM sodium phosphate buffer (pH 7.4) containing 0.5 M KCl, 0.5 M imidazole. After desalting the fractions with 10 mM sodium phosphate buffer (pH 7.0), 100 mM KCl using a HiTrap desalting column (GE Healthcare), the solution was applied to HiTrap Q HP. RNAP was eluted at a salt concentration of 0.4 M KCl. Fractions containing RNAP were applied to a Superdex 200 HR 10/30 column equilibrated with 10 mM sodium phosphate buffer (pH 7.0) containing 100 mM KCl.

### *In vitro* transcription assay.

A DNA fragment containing a region spanning 190 bp upstream and 232 bp downstream of the translational initiation codon of TK0252 was amplified from T. kodakarensis KOD1 genomic DNA using the primers PTK0252_F and PTK0252_R_2. Similarly, DNA fragments containing a region spanning 202 bp upstream and 230 bp downstream of the initiation codon of TK0271 and a region spanning 192 bp upstream and 251 bp downstream of the initiation codon of TK0261 were amplified using the primer sets PTK0271_F/PTK0271_R_2 and PTK0261_F/PTK0261_R_2, respectively. Amplified fragments were ligated in the HincII site of pUC18. The resulting plasmid was used as a template to amplify the DNA template for *in vitro* transcription. Amplified fragments were purified by gel extraction, phenol/chloroform/isoamyl alcohol treatment, and ethanol precipitation. For *in vitro* transcription reactions, template DNAs (50 nM) were incubated with 200 nM T. kodakarensis RNAP (TkRNAP), 300 nM TkTBP, 300 nM TkTFB, and 0 to 1,000 nM TK0271 protein in a reaction buffer (20 μl) consisting of 20 mM HEPES (pH 7.5), 11 mM magnesium acetate, 400 mM potassium acetate, 10 mM DTT, 6 mM ATP, 6 mM GTP, 6 mM CTP, 6 mM UTP, and 0.8 μl RNA secure (Thermo Fisher Scientific) at 90°C for 25 min. Effects of erythrose 4-phosphate on *in vitro* transcription were analyzed with 1,000 nM TK0271 protein and various concentrations of erythrose 4-phosphate from 0 to 2 mM. After the reactions, samples were treated with RNase-free DNase (Promega, Fitchburg, WI) at 37°C for 30 min to digest template DNA. Samples were mixed with the same volume of gel loading buffer II (Thermo Fisher Scientific) and incubated at 70°C for 3 min. Denatured RNA samples were loaded onto a 5% acrylamide denaturing gel containing 7.0 M urea. Gels were run in 1× TBE buffer at 100 V for 90 min. After electrophoresis, gels were stained with ethidium bromide. The fluorescence of probes intercalated with ethidium bromide was detected with a charge-coupled device camera (LumiVision PRO 400 EX; AISIN Seiki, Kariya, Japan) with lens filters (YA3 and DR655; Kenko Tokina, Tokyo, Japan).

### Construction of TK1227 and TK0271 gene disruption strains.

The TK1227 and TK0271 genes with 1,000 bp of their upstream and downstream regions were amplified by PCR using T. kodakarensis genomic DNA as a template and the primers DTK0271_F1 and DTK0271_R1 for TK0271 and DTK1227_F and DTK1227_R for TK1227. Amplified DNA fragments were digested with PstI and XbaI (for TK0271) or SalI (for TK1227) and subsequently inserted into pUD3 (35). The plasmids were used as templates for inverse PCR with the primers DTK0271_F2/DTK0271_R2 for TK0271 and ITK1227_F/ITK1227_R for TK1227 in order to remove the respective coding regions.

Transformation of T. kodakarensis was performed as previously described via single-crossover insertion and popout recombination (52). T. kodakarensis KU216 (31), which displays uracil auxotrophy, was used as a host along with the *pyrF* gene as the marker gene. Cells transformed with disruption vectors were cultivated in ASW-AA-S^0^ medium for 48 h at 85°C. Cells were cultivated again in the same medium to enrich transformants that display uracil prototrophy. Cells were then diluted with 0.8× ASW and spread onto solid ASW-AA medium supplemented with uracil and 5-FOA. Cells were grown for 2 days at 85°C until colonies were observed. Individual transformants were selected, and their genotypes were examined by PCR and DNA sequencing. The constructed ΔTK0271 and ΔTK1227 strains were designated KAR1 and KTY1, respectively.

### Complementation experiments on the TK0271 disruption strain.

The TK0271 gene (315 bp) and its promoter region (202 bp) were amplified by PCR using the primer set TK0271compF1/TK0271compR1. The primer annealing to the 3′ terminus of the coding region included the transcriptional terminator sequence of the glutamate dehydrogenase gene of Pyrococcus furiosus. The amplified fragment was digested with NotI and ApaI and subsequently ligated into the respective sites of pLC70, a shuttle vector that replicates autonomously in both E. coli and T. kodakarensis (33). The *trpE* gene lies between NotI and ApaI of pLC70 and thus is not present in the constructed plasmid, designated pTK0271comp. In addition, pLC70 was digested by NotI and ApaI, blunted, and self-ligated. The resulting plasmid, pLCΔtrpE, was used in control experiments. pTK0271comp or pLCΔtrpE was introduced into the ΔTK0271 strain using 3-hydroxy-3-methylglutaryl-coenzyme A (HMG-CoA) reductase as a selectable marker with previously reported procedures (32).

### Growth measurements.

Growth characteristics of the host strain (KU216) and mutant strains (KAR1 and KTY1) were measured as follows. Each strain was cultured in 20 ml of ASW-AA medium supplemented with 0.2% (wt/vol) S^0^, 0.5% (wt/vol) sodium pyruvate, 0.5% (wt/vol) maltodextrin, and 0.1 mM Na_2_WO_4_ and cultured at 85°C. When necessary, Trp was omitted from the medium. Cell density was monitored by measuring turbidity at 660 nm.

### Transcriptome analysis with DNA microarray.

T. kodakarensis KU216 was cultivated in 200 ml ASW-AA-Ura-S^0^-Pyr-Mdx medium in the presence and absence of Trp, Tyr, and Phe, and cells were harvested in the log phase (OD_660_ of 0.06 to 0.105). Total RNAs were extracted using an RNeasy Midi kit (Qiagen). Fluorescently labeled cDNA, used for hybridization, was prepared using the RNA fluorescence labeling core kit (TaKaRa Bio, Kusatsu, Japan). Total RNA was annealed with random hexamers, and reverse transcription was performed in solutions containing CyDye-labeled dUTP (Cy3-dUTP or Cy5-dUTP) (GE Healthcare). RNA was subsequently degraded with RNase H, and the labeled cDNA was purified using a column supplied by the manufacturer. The labeled cDNA was dissolved in hybridization buffer (30 μl) containing 6× SSC (0.9 M NaCl, 84 mM sodium citrate), 0.2% SDS, 5× Denhardt’s solution, and 0.1 mg/ml denatured salmon sperm DNA. Hybridization was performed under a coverslip (Spaced Cover Glass XL; TaKaRa Bio) in a humidity chamber at 65°C for 12 to 15 h. After hybridization, the microarray plates were washed four times with 2× SSC and 0.2% SDS at 55°C for 5 min, rinsed in 0.05× SSC, and dried by centrifugation. The fluorescence images derived from the Cy3 and Cy5 dyes were recorded using an Affymetrix 428 array scanner (Affymetrix, Santa Clara, CA). Microarray images were analyzed using ImaGene, version 5.5, software (Bio Discovery, Marina Del Ray, CA). The signal intensity of each spot was measured and normalized with the total signal intensities. The normalized signal intensities of individual spots (each representing a specific transcript) derived from cells grown under different cultivation conditions (i.e., with and without a particular amino acid) were compared.
