# Supplementary figures and images for: The TK0271 Protein Activates Transcription of Aromatic Amino Acid Biosynthesis Genes in the Hyperthermophilic Archaeon Thermococcus kodakarensis
Source: mBio. 2019 Sep 10;10(5):e01213-19. doi: 10.1128/mBio.01213-19 (PMC6737238; doi:10.1128/mBio.01213-19)

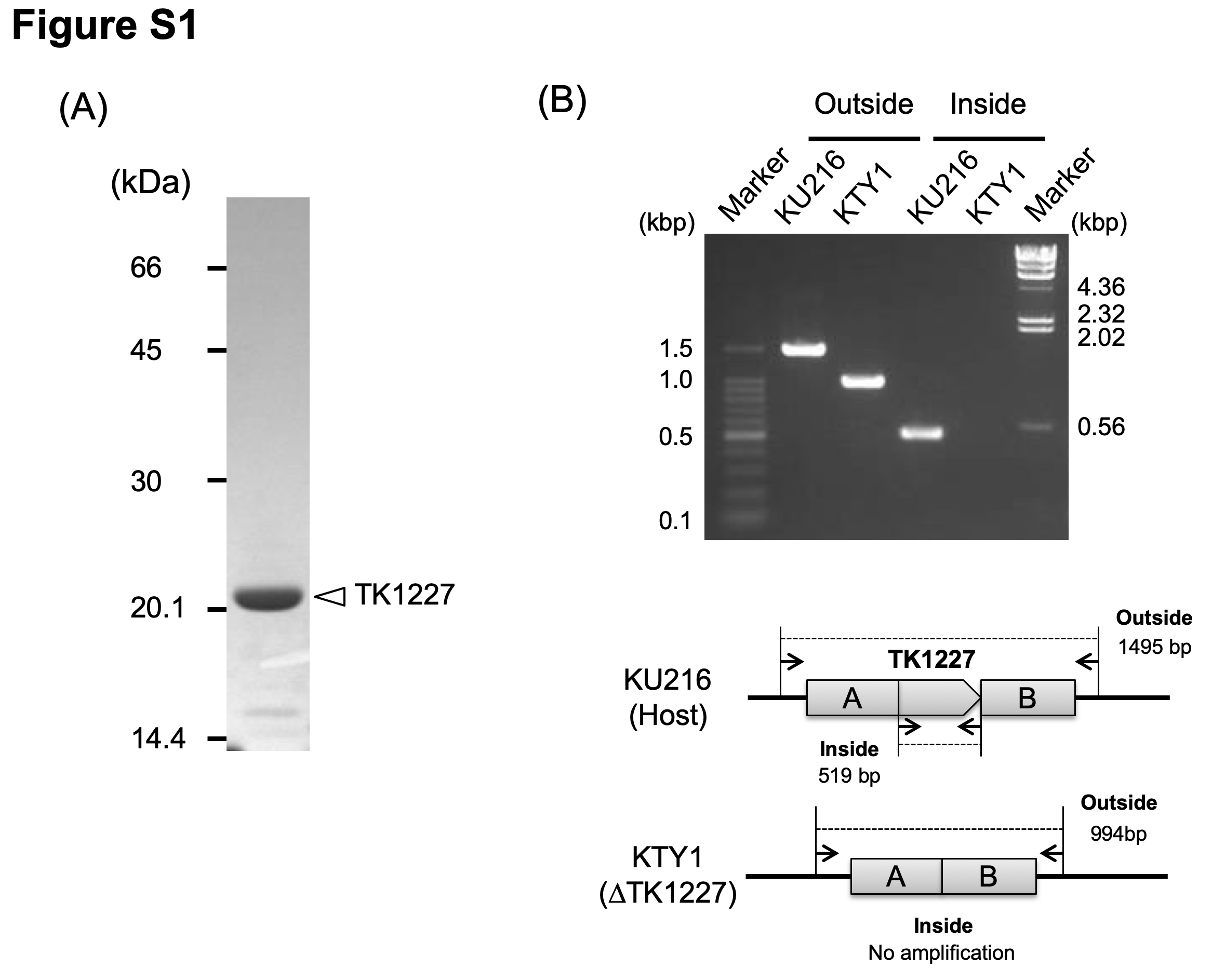

Supplement: FIG S1 [file mBio.01213-19-sf001.tif]

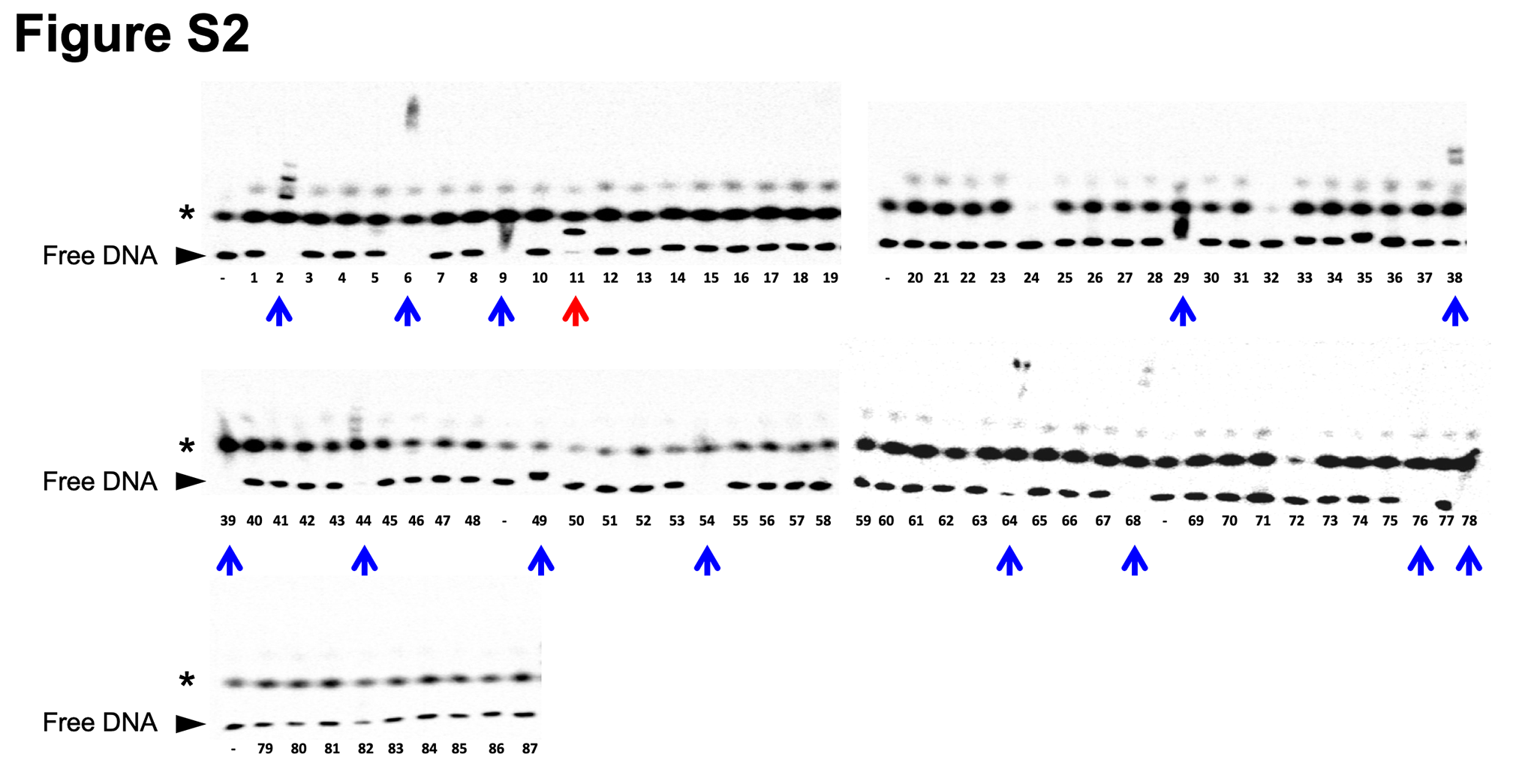

Supplement: FIG S2 [file mBio.01213-19-sf002.tif]

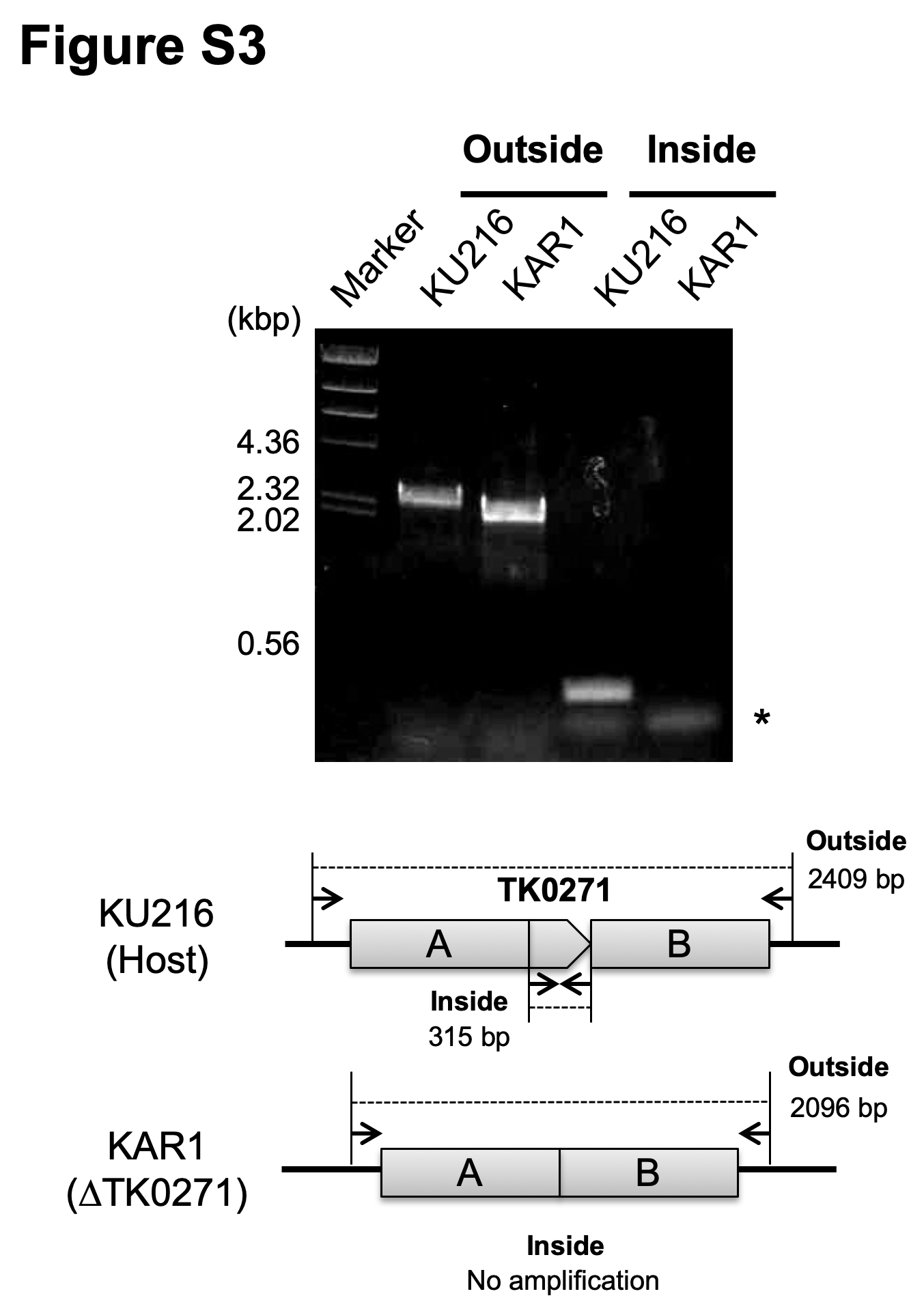

Supplement: FIG S3 [file mBio.01213-19-sf003.tif]

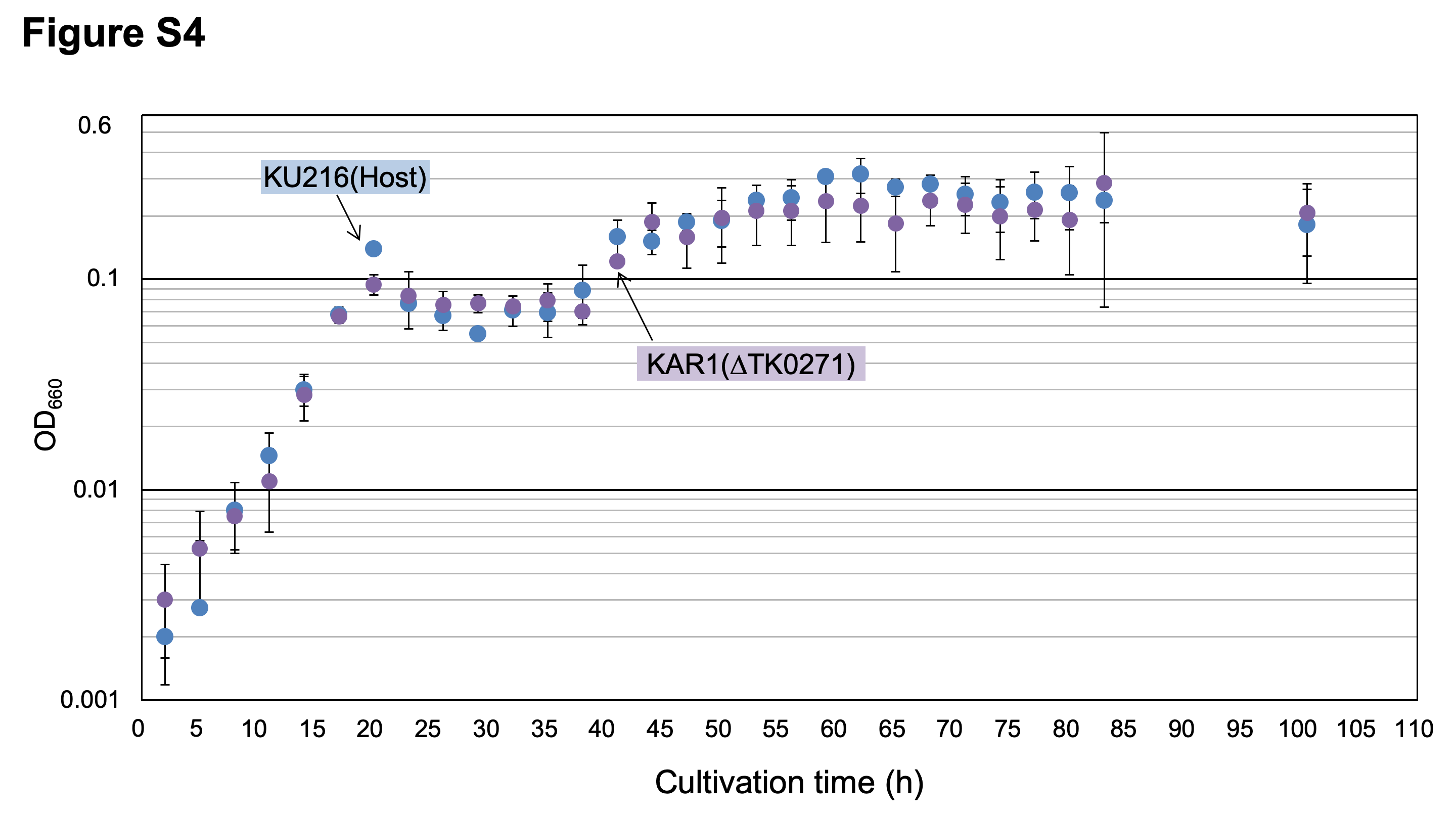

Supplement: FIG S4 [file mBio.01213-19-sf004.tif]

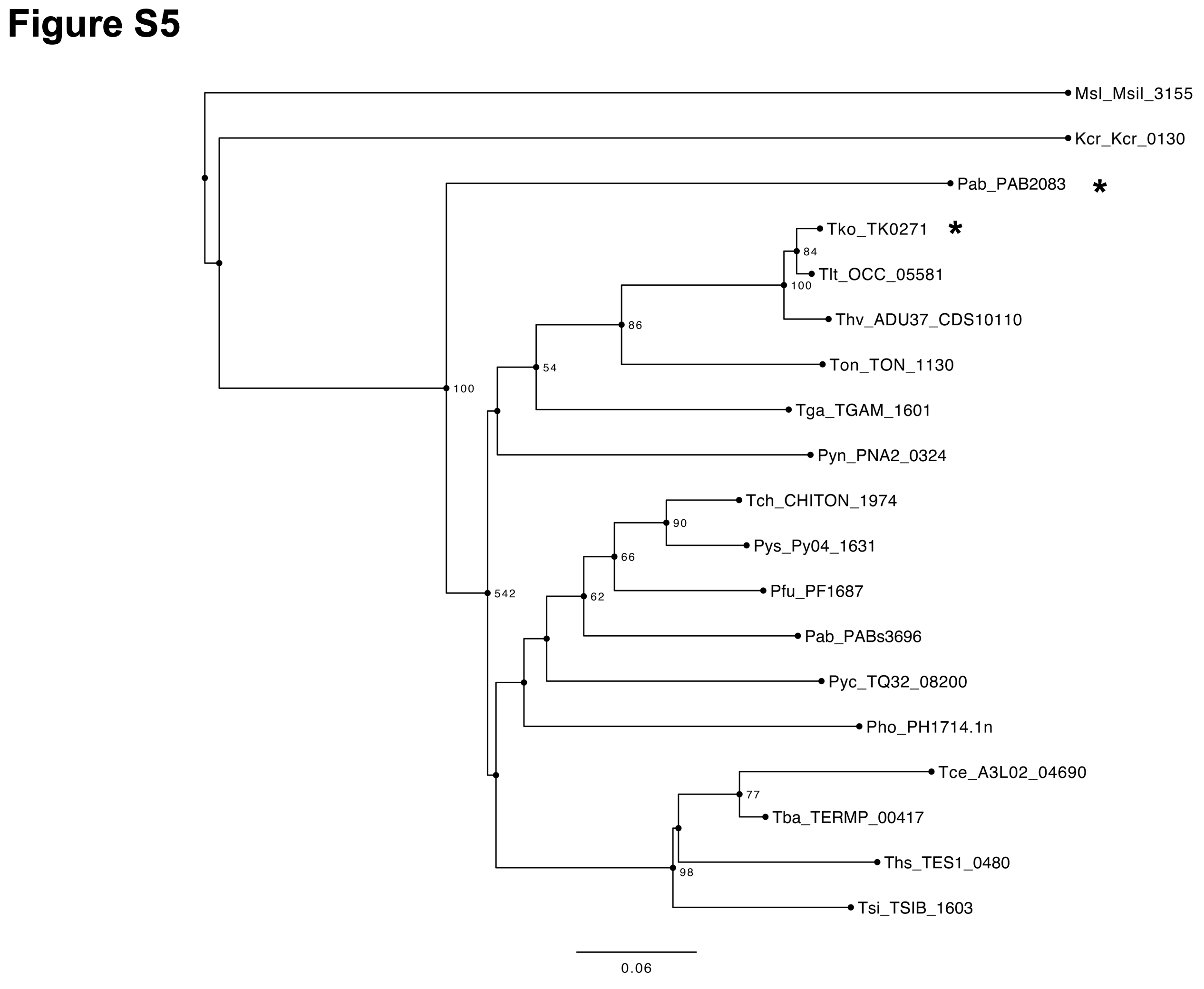

Supplement: FIG S5 [file mBio.01213-19-sf005.tif]

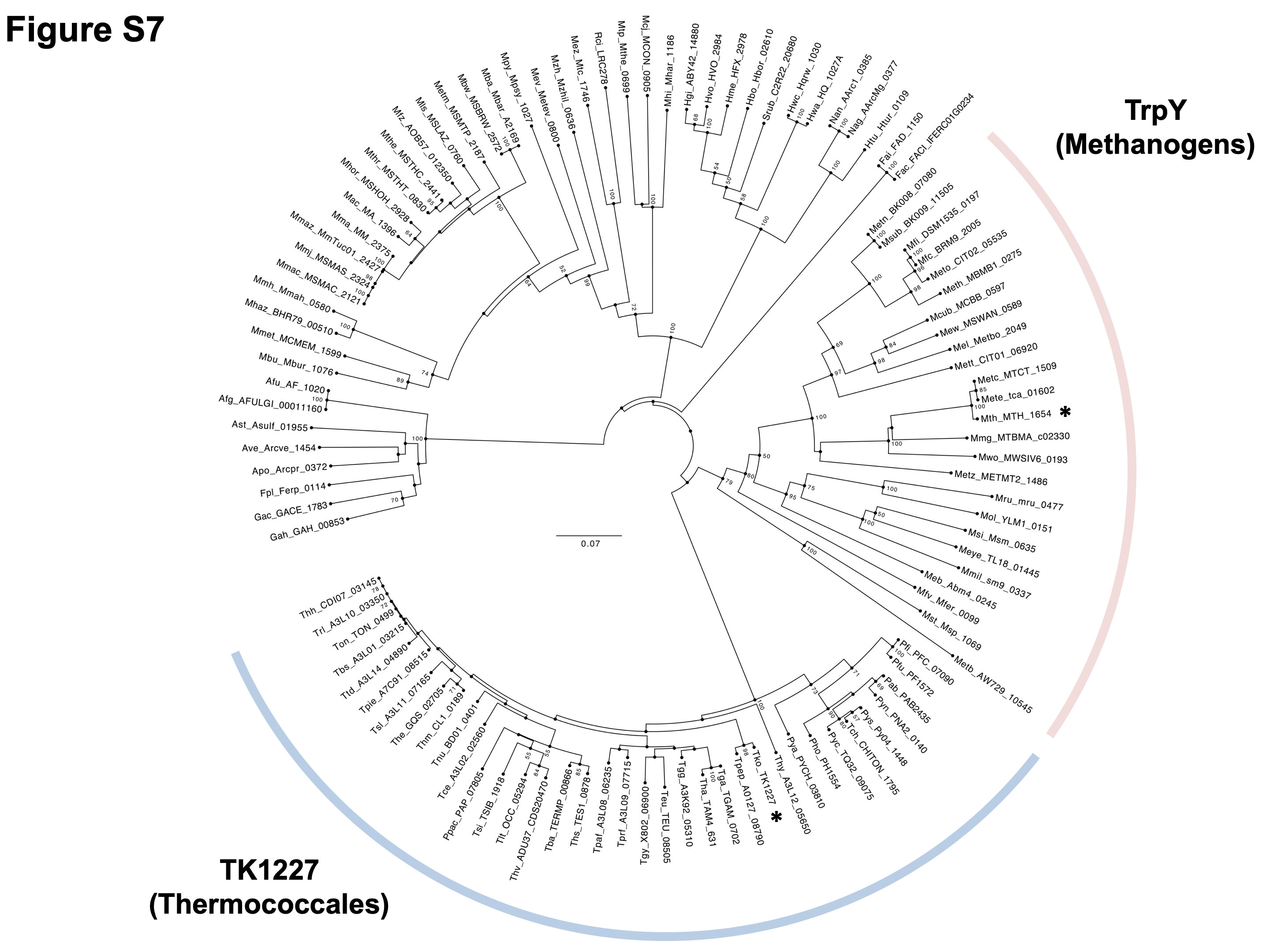

Supplement: FIG S7 [file mBio.01213-19-sf007.tif]
