# Supplementary material for: The TK0271 Protein Activates Transcription of Aromatic Amino Acid Biosynthesis Genes in the Hyperthermophilic Archaeon Thermococcus kodakarensis
Source: mBio. 2019 Sep 10;10(5):e01213-19. doi: 10.1128/mBio.01213-19 (PMC6737238; doi:10.1128/mBio.01213-19)
Supplement: FIG S6 [file mBio.01213-19-sf006.pdf]

Fig. S6

```
b0754_aroG      ---------------MNYQNDDLRIKEIKELLPPVALLEKFPATENAANTVAHARKAIHK
b1704_aroH      ----------------MNRTDELRTARIESLVTPAELALRYPVTPGVATHVTDSRRRIEK
b2601_aroF      --------------MQKDALNNVHITDEQVLMTPEQLKAAFPLSLQQEAQIADSRKSISD
YBR249C         MSESPMFAANGMPKVNQGAEEDVRILGYDPLASPALLQVQIPATPTSLETAKRGRREAID
YDR035W         ------MFIKNDHAGDRKRLEDWRIKGYDPLTPPDLLQHEFPISAKGEENIIKARDSVCD
TK0268          ------------------------------------------MRFKFEKGSKPKTVVKVGG
PF1690          --------------------------------------------MKYSKEYKEKTVVKIND
APE_0581.1      -----------------------------------MAGFKGVKLALKSEERRETVVEVEG

b0754_aroG      ILKGNDDRLLVVIGPCSIHDPVAAKEYATRLLALREELKDELEIVMRVYFEKPRTTVGWK
b1704_aroH      ILNGEDKRLLVIIGPCSIHDLTAAMEYATRLQSLRNQYQSRLEIVMRTYFEKPRTVVGWK
b2601_aroF      IIAGRDPRLLVVCGPCSIHDPETALEYARRFKALAAEVSDSLYLVMRVYFEKPRTTVGWK
YBR249C         IITGKDDRVLVIVGPCSIHDLEAAQEYALRLKKLSDELKGDLSIIMRAYLEKPRTTVGWK
YDR035W         ILNGKDDRLVIVIGPCSLHDPKAAYDYADRLAKISEKLSKDLLIIMRAYLEKPRTTVGWK
TK0268          VKIGDG--FTVIAGPCAVESQEQIMKVAEFLAEMGVKVLR--------------------
PF1690          VKFGEG--FTIIAGPCSIESRDQIMKVAEFLAEVGIKVLR--------------------
APE_0581.1      VRIGGGS-KAVIAGPCSVESWEQVREAALAVKEAGAHMLR--------------------
                   *         ***           *

b0754_aroG      GLINDPHMDNSFQINDGLRIARKLLLDINDSGLPAAGEFLDMITPQYLADLMSWGAIGAR
b1704_aroH      GLISDPDLNGSYRVNHGLELARKLLLQVNELGVPTATEFLDMVTGQFIADLISWGAIGAR
b2601_aroF      GLINDPHMDGSFDVEAGLQIARKLLLELVNMGLPLATEALDPNSPQYLGDLFSWSAIGAR
YBR249C         GLINDPDVNNTFNINKGLQSARQLFVNLTNIGLPIGSEMLDTISPQYLADLVSFGAIGAR
YDR035W         GLINDPDMNNSFQINKGLRISREMFIKLVEK-LPIAGEMLDTISPQFLSDCFSLGAIGAR
TK0268          GGAFKPRTSPYSFQGHGEEALKWMRRAADEYGLVTVTEVMDVSQVELVAKYSDMLQIGAR
PF1690          GGAFKPRTSPYSFQGYGEKALRWMREAADEYGLVTVTEVMDTRHVELVAKYSDILQIGAR
APE_0581.1      GGAFKPRTSPYSFQGLGLEGLKLLRRAGDEAGLPVVTEVLDPRHVETVSRYADMLQIGAR
                *    *          *                    *  *                ****

b0754_aroG      TTESQVHRELASGLSCPVGFKNGTDG-TIKVAIDAINAAGAPHCFLSVTKWGHSAIVNTS
b1704_aroH      TTESQIHREMASALSCPVGFKNGTDG-NTRIAVDAIRAARASHMFLSPDKNGQMTIYQTS
b2601_aroF      TTESQTHREMASGLSMPVGFKNGTDG-SLATAINAMRAAAQPHRFVGINQAGQVALLQTQ
YBR249C         TTESQLHRELASGLSFPVGFKNGTDG-TLNVAVDACQAAAHSHHFMGVTKHGVAAITTTK
YDR035W         TTESQLHRELASGLSFPIGFKNGTDG-GLQVAIDAMRAAAHEHYFLSVTKPGVTAIVGTE
TK0268          NSQNFELLKAVGKVDNPVVLKRGMANTVQELLYSAEYILSGGNENVILCERGIRTFETST
PF1690          NSQNFELLKEVGKVENPVLLKRGMGNTIQELLYSAEYIMAQGNENVILCERGIRTFETAT
APE_0581.1      NMQNFPLLREVGRSGKPVLLKRGFGNTVEELLAAAEYILLEGNWQVVLVERGIRTFEPST
                                *   * *           *                *

b0754_aroG      GNGDCHIILRGGKE-PNYSAKHVAEVKEGLNKAGLP----AQVMIDFSHANSSKQFKKQM
b1704_aroH      GNPYGHIIMRGGKK-PNYHADDIAACDTLHEFDLP----EHLVVDFSHGNCQKQHRRQL
b2601_aroF      GNPDGHVILRGGKA-PNYSPADVAQCEKEMEQAGLR----PSLMVDCSHGNSNKDYRRQP
YBR249C         GNEHCFVILRGGKKGTNYDAKSVAEAKAQLPAG------SNGLMIDYSHGNSNKDFRNQP
YDR035W         GNKDTFLILRGGKNGTNFDKESVQNTKKQLEKAGLTDDSQKRIMIDCSHGNSNKDFKNQP
TK0268          RFTLDISAVPVVKELSHLPIIVDPSHPAGRRELVIP--LAKAAYAVGADGIMVEVHPEPD
PF1690          RFTLDISAVPVVKELSHLPIIVDPSHPAGRRSLVIP--LAKAAYAIGADGIMVEVHPEPE
APE_0581.1      RFTLDVAAVAVLKEATHLPVIVDPSHPAGRRSLVPA--LAKAGLAAGADGLIVEVHPNPE
                            *

b0754_aroG      DVCADVCQQIAGGEKAIIGVMVESHLVEGNQSL--ESGE-PLAYGKSITDACIGWEDTDA
b1704_aroH      EVCEDICQQIRNGSTAIAGIMAESFLREGTQKI--VGSQ-PLTYGQSITDPCLGWEDTER
b2601_aroF      AVAESVVAQIKDGNRSIIGLMIESNIHEGNQSS--EQPRSEMKYGVSVTDACISWEMTDA
YBR249C         KVNDVVCEQIANGENAITGVMIESNINEGNQGIPAEG-KAGLKYGVSITDACIGWETTED
YDR035W         KVAKCIYDQLTEGENSLCGVMIESNINEGRQDIPKEGGREGLKYGCSVTDACIGWESTEQ
TK0268          KALSDSAQQLTFEDFERLLGELEGLGWKGTTSR--EAAEGVPRYVLSERVEENDWYTEVF
PF1690          KALSDSQQQLTFDDFLQLLKELEALGWKG-------------------------------
APE_0581.1      EALSDAKQQLTPGEFARLMGELRWHRLL--------------------------------
                        *

b0754_aroG      LLRQLANAVKARRG----
b1704_aroH      LVEKLASAVDTRF-----
b2601_aroF      LLREIHQDLNGQLTARVA
YBR249C         VLRKLAAAVRQRREVNKK
YDR035W         VLELLAEGVRNRRKALKK
TK0268          LMELGGLGWRA-------
PF1690          ------------------
APE_0581.1      ------------------
```
